# Barriers and facilitators to the implementation of a paediatric palliative care team

**DOI:** 10.1186/s12904-018-0274-8

**Published:** 2018-02-12

**Authors:** Lisa M. Verberne, Marijke C. Kars, Sasja A. Schepers, Antoinette Y. N. Schouten-van Meeteren, Martha A. Grootenhuis, Johannes J. M. van Delden

**Affiliations:** 1 Department of Medical Humanities, Julius Center for Health Sciences and Primary Care, University Medical Center Utrecht, PO BOX 85500, Heidelberglaan 100, 3508 GA Utrecht, The Netherlands; 2 Psychosocial Department, Emma Children’s Hospital, Academic Medical Center, Meibergdreef 9, 1105 AZ Amsterdam, The Netherlands; 3 Princess Máxima Center for Pediatric Oncology, Lundlaan 6, 3584 AE Utrecht, The Netherlands; 4 Department of Psychology, St. Jude Children’s Research Hospital, 262 Danny Thomas Place, Memphis, TN 38105 USA; 5 Department of Pediatric Oncology, Emma Children’s Hospital, Academic Medical Center, Meibergdreef 9, 1105 AZ Amsterdam, The Netherlands

**Keywords:** Paediatrics, Palliative care, Paediatric palliative care team, Implementation, Healthcare professional, Barrier, Facilitator

## Abstract

**Background:**

Over the last decade, paediatric palliative care teams (PPCTs) have been introduced to support children with life-limiting diseases and their families and to ensure continuity, coordination and quality of paediatric palliative care (PPC). However, implementing a PPCT into an organisation is a challenge. The objective of this study was to identify barriers and facilitators reported by healthcare professionals (HCPs) in primary, secondary or tertiary care for implementing a newly initiated multidisciplinary PPCT to bridge the gap between hospital and home.

**Methods:**

The Measurement Instrument for Determinants of Innovations (MIDI) was used to assess responses of 71 HCPs providing PPC to one or more of the 129 children included in a pilot study of a PPCT based at a university children’s hospital. The MIDI (29 items) assessed barriers and facilitators to implementing the PPCT by using a 5-point scale (completely disagree to completely agree) and additional open-ended questions. Items to which ≥20% of participants responded with ‘totally disagree/disagree’ and ≥80% responded with ‘agree/totally agree’ were considered as barriers and facilitators, respectively. A general inductive approach was used for open-ended questions.

**Results:**

Reported barriers to implementing a PPCT were related to the HCP’s own organisation (e.g., no working arrangements related to use of the intervention [PPCT] registered, other organisational changes such as merger going on). Reported facilitators were mainly related to the intervention (correctness, simplicity, observability and relevancy) and the user scale (positive outcome expectations, patient satisfaction) and only once to the organisation scale (information accessibility). Additionally, HCPs expressed the need for clarity about tasks of the PPCT and reported having made a transition from feeling threatened by the PPCT to satisfaction about the PPCT.

**Conclusion:**

Positive experiences with the PPCT are a major facilitator for implementing a PPCT. Tailored organisational strategies such as working arrangements by management, concrete information about the PPCT itself and the type of support provided by the PPCT should be clearly communicated to involved HCPs to increase awareness about benefits of the PPCT and ensure a successful implementation. New PPCTs need protection and resources in their initial year to develop into experienced and qualified PPCTs.

**Electronic supplementary material:**

The online version of this article (10.1186/s12904-018-0274-8) contains supplementary material, which is available to authorized users.

## Background

Paediatric palliative care (PPC) can improve the quality of life of children with life-limiting or life-threatening diseases and their families [1]. Healthcare professionals (HCPs) often find it challenging to initiate and provide adequate PPC. Common barriers to providing PPC include uncertain prognosis, time limitations, discomfort with providing palliative care and lack of knowledge, experience, education or staff support [2–8]. Additional barriers include lack of continuity and coordination of care because of the involvement of many professionals from healthcare, social care and education [3, 6, 7, 9–11]. The lack of adequate PPC can often give parents the feeling of being alone and having to provide this care themselves, particularly in home-based settings [12–15]. To improve the quality of PPC, the American Academy of Pediatrics (AAP), the Institute of Medicine (IoM) and the International Meeting for Palliative Care in Children, Trento (IMPaCCT), a task force of the European Association of Palliative Care, recommended the introduction of paediatric palliative care teams (PPCTs) [11, 14, 16]. Over the last decade, PPCTs have been introduced in several countries [2, 17–23].

In June 2012, a three-year pilot study with the first Dutch hospital–based PPCT was initiated at the Emma Children’s Hospital, Academic Medical Centre (AMC), Amsterdam. Our PPCT, like other PPCTs, operates in addition to paediatric and palliative care support that is already provided to patients and families. It aims to support children with a life-limiting disease and their families by ensuring continuity, coordination and high quality of PPC [2, 18–24]. Multidisciplinary teams provide continuous support throughout the patient’s disease trajectory, including around-the-clock availability for care consultation and bereavement care [18, 20–22, 24]. Some PPCTs also strengthen regular care at home by educating other involved HCPs [2, 18, 24].

Implementation of new services such as a PPCT can be influenced by various factors associated with the intervention itself (e.g., simplicity and compatibility), the user (e.g., knowledge and personal benefits), the organisation (e.g., time availability and performance feedback) and the socio-political context (e.g., regulations) [25–27]. To date, only some barriers and challenges to implementing PPCTs in practice have been identified. Toce et al. [18] described programmatic challenges related to societal and cultural norms or insufficient staffing when implementing and institutionalizing the FOOTPRINTS^SM^ program and attempted to overcome them by using educational and communicational strategies. Edlynn et al. [28] reported that many physicians do not use the PPCT because they consider that the family is not ready for PPC. Moreover, there is often an initial period of quiescence, followed by a rapid increase in the number of patients referred to a PPCT [29]. Key features to ensuring the rapid and early influx of referrals to the PPCT include formation of a special committee, an educational campaign before program implementation, focusing efforts on clinicians who refer in early weeks of a new PPCT and a plan for team growth [29].

Despite a well-developed project plan, sufficient financial and other resources, establishment in a large children’s hospital and a thoroughly built team of enthusiastic and skilled HCPs, our PPCT encountered some resistance from regular HCPs during the pilot. Specifically, the PPCT found it difficult to fill the gaps identified in PPC and to position the team as a meaningful and acceptable addition to the existing healthcare setting. To address these challenges, it was essential to identify barriers and facilitators to successfully implementing a PPCT from the perspective of other involved HCPs. To develop tailored implementation strategies for improving PPC in the intramural and home settings [26, 27, 30], in this study we identified HCP-reported barriers and facilitators to implementing the PPCT.

## Methods

### Study design and participants

A cross-sectional study was performed among HCPs working at the Emma Children’s Hospital or providing primary or secondary care for a child with life-limiting disease. Inclusion criteria for HCPs were (1) provision of PPC to one or more of the 129 children included in the three-year pilot of the PPCT based at the Emma’s Children Hospital, and (2) availability of a personal work-related email address. The 129 children included in the PPCT pilot were 0–18 years old, diagnosed with malignant or non-malignant life-limiting diseases, were in different phases of their palliative trajectory and were primarily cared for at home.

### Setting

In the Netherlands, the ambition is to enable children with a life-limiting disease to reside at home as much as possible. Children are admitted to a hospital only when the disease or its complications need to be treated. The main treating clinician in the tertiary hospital is responsible for providing adequate PPC for children at the hospital or at home. PPC is provided by primary (e.g., general practitioners or homecare organisations), secondary and tertiary HCPs. Paramedics and psychosocial professionals are also often involved in PPC. As most HCPs have limited experience in providing PPC, the newly initiated PPCT is added to this regular care setting. The PPCT aims to support regular HCPs in their duties and not to replace regular HCPs or other care structures. The PPCT also provides support to the child and family, irrespective of where the child receives care. The PPCT also works to bridge gaps between the home and hospital.

### Intervention

In June 2012, a three-year pilot study of the first Dutch PPCT was implemented at Emma Children’s Hospital. Given the diversity of its tasks, the involvement of various participants and organisational structures and the provision of support tailored to the specific needs of the child and family, the PPCT was considered a complex medical intervention as defined by the Medical Research Council [31]. The PPCT was designed and developed as per the recommendations of the AAP, IoM and IMPaCCT [11, 14, 16]. The PPCT is a multidisciplinary team comprising a core team of five specialised paediatric nurses (case managers) trained and experienced in PPC and a flexible shell of two child life specialists, one psychologist, one social worker and one chaplain. In addition, two paediatricians and two paediatric oncologists are assigned for regular consultation. The core team of nurses is responsible for the coordination, continuity and quality of PPC, irrespective of the child’s place of residence. By working proactively, they seek to avoid acute demands for support. The PPCT provides continuous support throughout the disease trajectory, including 24-h availability for consultation and bereavement care. The PPCT navigates parents through complex care processes and bridges the gap between home and hospital by communicating through regular phone calls, emails or personal visits at home and during hospitalisations. The PPCT also strengthens regular care at home by providing education and coaching on the job to other involved HCPs (e.g., homecare nurses or general practitioners). If regular care is not successful, the PPCT is qualified to take over the care by providing temporary nursing care at home. Further, the PPCT organizes weekly multidisciplinary conferences to promote discussions among patients and maximize the exchange of knowledge related to palliative care. These meetings also help determine the roles and activities of team members in supporting the child and family.

### Data collection

All eligible HCPs were invited by email to participate in the study. They were requested to complete an online survey via a direct link to the website www.hetklikt.nu to assess barriers and facilitators to implementing the PPCT. Evaluation of the PPCT from the parents’ perspective has been previously reported [32]. Two reminder e-mails were sent at 2-week intervals to all non-responders. Data were collected in September and October 2015, shortly after the three-year pilot study of the PPCT. The study was approved by the research ethics committee of the Academic Medical Centre, Amsterdam (July 15, 2015; reference number W15_183 # 15.0220). Informed consent was obtained online from all participating HCPs.

### Questionnaire

We used the evidence-based Measurement Instrument for Determinants of Innovations (MIDI) developed by Fleuren et al. [25, 26, 33] to identify the factors influencing the use of an intervention and help design new implementation strategies. The MIDI comprises four scales measuring determinants associated with the innovation (7 items), the user (11 items), the organisation (10 items) and the socio-political context (1 item). The questionnaire consists of 29 questions. The response scales range from 1 (‘totally disagree’) to 5 (‘totally agree’). In accordance with regulations of the MIDI questionnaire, we made some adjustments to the MIDI [33] after consulting with the project leader of the PPCT pilot and with Dr. M. Fleuren. We removed from the user scale question 13 about social support and from the organisation scale question 21 about staff capacity and question 25 about the coordinator, because most HCPs (e.g., general practitioners) worked independently and as single HCPs or only with few colleagues. From the organisation scale, we also excluded question 22 about financial resources and question 24 about material resources and facilities, because no appeal was made for financial or material resources for HCPs when collaborating with the PPCT. For question 8 of the user scale about personal benefits from involvement of the PPCT, we added an open-ended option ‘Otherwise, namely…’. Further, two open-ended questions were added at the end of the MIDI: ‘Do you have any tips or suggestions for improvements for the PPCT?’ and ‘If you have any comments, please describe them here’. In our sample, the internal consistency of the MIDI questionnaire was good–excellent, except for the organisation scale for which the internal consistency was sufficient (total scale: α = .93, innovation scale: α = .86, user scale: α = .90, organisation scale: α = .61) [34, 35]. In addition to the MIDI, background information such as age, gender, profession and years of experience in current profession was requested from responders.

### Statistical analyses

The Windows version of the Statistical Package for Social Sciences (SPSS) 21.0 was used for data analyses. Descriptive statistics (mean, standard deviation, range and percentage) were used to evaluate HCP-reported barriers and facilitators to implementing the PPCT, as indicated on the MIDI. After consulting with Dr. M. Fleuren, we decided that MIDI items to which ≥20% of participants responded with ‘totally disagree/disagree’ were considered barriers and those to which ≥80% of participants responded with ‘agree/totally agree’ were considered facilitators for implementing the PPCT. A general inductive approach was used for open-ended questions [36].

## Results

### Participants

Of the 207 HCPs eligible for participation, 71 completed the MIDI questionnaire (response rate 34.3%). HCPs were mainly doctors or nurses. The remaining participating HCPs were from a wide range of professions (e.g., paramedics or psychosocial care) and reflected the child-centred approach of PPC. Table 1 gives the characteristics of participants. HCPs who did not participate were doctors (*n* = 56), nurses (*n* = 32), paramedics (*n* = 20), psychosocial professionals (*n* = 17) and other professionals (*n* = 11).

**Table 1:** Characteristics of healthcare professionals participating in the study (*n* = 71)

	Doctors	Nurses	Paramedics^a^	Psychosocial professionals	Other	Total
	(*n* = 20)	(*n* = 33)	(*n* = 7)	(*n* = 9)	(*n* = 2)	(*n* = 71)
Gender (*n*; %)						
Male	4 (5.6)	1 (1.4)	1 (1.4)	0 (0)	0 (0)	6 (8.4)
Female	16 (22.5)	32 (45.1)	6 (8.4)	9 (12.7)	2 (2.8)	65 (91.5)
Mean age (range; years)	47.8 (39–59)	46.3 (27–62)	44.4 (28–59)	45.3 (29–57)	56.5 (49–64)	46.7 (27–64)
Mean duration at current job (range; years)	14.4 (1–30)	12.3 (5–30)	17.0 (4–30)	10.1 (1.5–25)	15.0 (15–15)^b^	13.1 (0.5–30)
Number of children supported for palliative care in past 5 years (*n*)						
≤ 2	12	8	3	4	1	28
3–5	7	10	1	2	0	20
6–9	0	9	1	0	1	11
≥ 10	1	6	2	3	0	12

^a^Paramedics comprised physiotherapists, ergotherapy professionals and professionals for visually impaired children

^b^Mean duration for current job missing for one participant

### Barriers and facilitators to implementing the PPCT

An overview of HCP-reported factors that influence use of the PPCT is given in Additional file 1: Table S1. Two barriers and 15 facilitators to implementing a PPCT were identified.

The two perceived barriers to implementing a PPCT were related to the organisational scale, namely ‘no formal ratification of the intervention by management’ (54% of HCPs reported that there were no working arrangements related to use of the intervention [PPCT]) and ‘unsettled organisation’ (28% of HCPs reported other organisational changes such as reorganisation or merger or other innovations going on).

Facilitators to implementing a PPCT were mainly related to the intervention and user scales. Facilitators in the intervention scale included ‘correctness’ (82% reported that the way the PPCT operates is based on factually correct knowledge of PPC), ‘simplicity’ (92% found the PPCT not too complex to use), ‘observability’ (80% said the outcomes of using the PPCT are clearly observable) and ‘relevance for the patient’ (87% reported the PPCT as relevant for their patients). Facilitators in the user scale included five ‘outcome expectations’: 83% of HCPs expected the care to be better attuned to needs of the child and parents due to PCCT involvement, 83% expected the PPCT to improve the quality of care, 80% believed that the PPCT helps improve the continuity of care, 87% expected parents not to feel abandoned when the PPCT is involved and 82% expected that the PPCT helps parents to continue caregiving at home. Other reported facilitators to implementing a PPCT in the user scale were a ‘personal benefit’ (83% reported that the PPCT supports them in providing PPC), the ‘professional obligation’ (90% felt that collaborating with the PPCT was an obligation), ‘patient satisfaction’ (83% believed that their patients/parents will be satisfied when using the PPCT), ‘descriptive norm’ (96% reported that more than half to all colleagues in their own work field will work together with the PPCT) and the ‘motivation to comply’ (87% found the opinion of patients/parents about the PPCT important). The only facilitator in the organisation scale was ‘information accessibility’: 80% of the HCPs reported that they easily received information about patients/parents from the PPCT.

Responses to the three open-ended questions (Table 2) revealed that HCPs (1) expressed a need for more clarity about possible tasks, responsibilities and commitment of the PPCT itself, and the need for a precise alignment thereof with parents and other HCPs involved; (2) expressed having made a transition from feeling threatened by the PPCT towards satisfaction about the PPCT; and (3) valued the PPCT members’ expertise and appreciated that the PPCTs kept active direction while giving other involved HCPs enough space for their own initiatives. One HCP reported that because of the PPCT’s involvement and inadequate advice, she and the patient’s parents became frustrated and cautioned about the involvement of too many HCPs.

**Table 2:** Illustrative responses of healthcare professionals to three open-ended questions

Question	Quote
Personal benefit or drawback due to the PPCT’s involvement	#10 General practitioner: *Especially regarding a child with a malignant diagnosis not too many health care professionals should be involved. My experience was that the PPCT nurse caused unrest and irritation with the parents and with me, and provided inadequate advice on medication.*
	#27 Paediatric nurse: *It is important for those involved to clearly agree who has which tasks at the start of the collaboration and to communicate with the family how the involvement is tuned.*
	#35 Paediatric nurse: *It is good that the PPCT exists, they keep control, there is enough space for your own input and consultation.*
	#41 Ergotherapy professional: *This year the tasks/responsibilities (of the PPCT) have become more evident. In the past, a lot was unclear whereby tasks that are in my opinion my responsibility were also done by the PPCT, resulting in discord.*
	#69 Paediatric oncologist: *It helps if the case manager is also trained on paediatric oncology knowledge to guide these children.*
Improvements for PPCT	#8 Paediatric nurse: *Feedback can absolutely be improved.*
	#11 Professional specialised in the development of visually impaired children: *Slightly faster clarification on how the contact will be when a child dies. Is there a final meeting? Now parents have to organise this themselves if they want it. It could also be arranged by the PPCT.*
	#14 Ergotherapy professional: *Collaboration between ergotherapy and PPCT was difficult at first. Formal requests were started up by PPCT members without involvement of an ergotherapy professional. This process has improved. The lines of communication are now shorter.*
	#23 Paediatric nurse: *I have no tips or suggestions for improvement. In the beginning we were sceptical towards the PPCT, perhaps afraid that they (PPCT) would take over our work.*
	#39 Paediatric nurse: *Continue with what the PPCT does and try to become of even more value for both organisations and the client. Increase publicity of what you can do.*
	#58 Social worker: *I prefer to be directly and regularly informed when any changes occur within the tasks of the PPCT.*
Comments related to the PPCT	#2 Paediatric nurse: *I think that for many parents and children who are dealing with several doctors, it is very pleasant to have one coordinator.*
	#4 Paediatric oncologist: *In the collaboration it is inevitable that the moments with patients and parents decrease. I find that a pity but nobody can do something about it.*
	#5 Paediatric physiotherapist: *I think it is nice when more is known within our practice about the possibilities of collaboration with the PPCT.*
	#40 Paediatric physiotherapist: *The intervention seems to me very important for parents. I will keep in mind that I can also call in the PPCT. I did not realise that.*
	#59 Paediatric nurse: *I am very satisfied about the collaboration with the PPCT. It has many advantages. Especially the short lines of communication with oncologists, as a result of which they think along to come up with solutions for certain questions about pain, sedation, etc.*
	#71 Paediatric nurse: *Continue like this and utilise each other’s qualities, certainly do not take over. I prefer to keep this care. And I notice that there is fear of losing the PPCT.*

*PPCT* paediatric palliative care team

## Discussion

This is the first Dutch study to identify factors that influence the implementation of a new PPCT, aimed at bridging the gap between home and hospital and ensuring high quality in the delivery of PPC. Organisational changes within the HCPs’ own organisation and the lack of working arrangements for collaborating with the PPCT were the two barriers reported by HCPs. The 15 facilitators to implementing the PPCT were mainly intervention and user related. Further analyses revealed that these facilitators reflected the perception of HCPs that the PPCT is relevant for the child and family, the quality of PPC improves because of the PPCT’s involvement and that the PPCT itself is an intervention that is not too complex to use. These perceptions are motivators for HCPs to collaborate with the PPCT.

The number of barriers to implementing a PPCT identified in this study were fewer than the number of facilitators. A possible explanation is that HCPs consider the PPCT to be a useful addition to PPC in which they experience no or few barriers. However, the PPCT also felt some resistance from other HCPs involved in PPC, which might be explained by the fact that members of the PPCT were sufficiently aware of being entrants to regular PPC and they found adequate strategies to collaborate with other involved HCPs. Another explanation is that by asking HCPs about barriers and facilitators at the end of the pilot study, the positive experiences of HCPs with the PPCT have overcome the possible barriers. For example, experiencing that the PPCT is relevant to the child and parents, that the collaboration with the PPCT goes smoothly and that the quality and continuity of PPC improves can stimulate an open attitude of HCPs to the implemented PPCT.

The identified facilitators also revealed that the most important factor for establishing a PPCT is that members demonstrate what they add to patient care for HCPs. Therefore, the value of the PPCT for HCPs seems to mainly turn out in practice. In other words, positive experiences with the PPCT also work as an important facilitator for its successful implementation. These findings imply that new PPCTs might often have a difficult initial phase as it takes time for positive experiences to establish, which was reflected in some qualitative answers for the open-ended questions. Newly introduced PPCTs should prepare for this difficult initial phase by incorporating time and space for other HCPs to experience their value and providing increased visibility. PPCT members should also have the perseverance to survive this phase. Future research can investigate whether and how barriers and facilitators to implementing a PPCT change over time by comparing outcomes of the MIDI performed shortly after start of the PPCT with those performed two years later. Additionally, an evaluation of whether certain implementation strategies are effective is also recommended.

The PPCT is likely important for not only parents [32] but also HCPs. Given its expertise and experience, the PPCT can train and support HCPs, which can help HCPs feel empowered in performing their role within their professional domain. Therefore, the dual approach of the PPCT in being supportive to both parents and HCPs is important to its success.

Our study showed that the role of the PPCT should be clarified and delineated for HCPs. Although the PPCT strives to not replace regular HCPs but instead build a strong care network around the child and family by involving regular HCPs, it is important that they share this goal and agree on role delineation. In addition to building relationships based on trust, agreements on responsibilities as well as sharing information in service of the child and the families should be made. To facilitate role clarification of the PPCT, concrete information about the support and responsibilities of the PPCT need to be provided through information booklets, an up-to-date website and during PPC educational sessions. Also, clear working arrangements between the PPCT and HCPs at the start of their collaboration can promote successful implementation of the PPCT.

Although studies by Gans et al. and Arland et al. [37, 38] indicate that the number of hospitalisations decrease with a PPCT, almost half (46.5%) of responding HCPs did not agree with this statement in the MIDI questionnaire. In addition, 38% of HCPs in this study did not report that the PPCT helped with proactive care planning throughout the disease trajectory. Although the IoM recommends advance care planning in early phases of the palliative process, in our opinion the PPCT can still make some progress, for instance, by improving its skills on advance care planning and providing better education and guidance for involved HCPs in advance care planning [11].

This study had several strengths and limitations. Most Dutch HCPs have a personal work-related email address for daily use, even when they are employed in a large organisation. As such, we assume that the likelihood for bias related to use of a personal work-related email addresses is limited. The online design of data collection was chosen because Dutch HCPs are well versed in information technology. In addition, specific tools in the online system for completing the web-based survey could be used to prevent non-response to items. Despite the advantage of an online survey wherein respondents can complete the questionnaire at their convenience, which might increase the likelihood of participation [39], the response rate of 34% was low. The limited response rate might be because HCPs with a more positive attitude towards the PPCT and HCPs who were more often involved in PPC were more likely to participate in the survey. Nonetheless, when compared with other web-based surveys, the response rate of our study lies within the normal ranges of 20%–47% [40]. We cannot accurately determine whether and how the limited response rate affected study results, because detailed characteristics of non-responders were unknown. Future research could focus on potential differences between specific HCP subgroups. Most of our participants were females, which is consistent with the Dutch scenario in which mainly female HCPs are involved in PPC. The reliability score was lower for the organisation scale than for other and total scales of the MIDI, probably because the researchers excluded several questions in the organisation scale as they did not fit in a PPCT setting. It should be noted that MIDI outcomes that did not qualify as barriers or facilitators could still have had a substantial influence on the implementation process of the PPCT. Several items came close to the cut offs set for facilitators (≥80% agreement) or barriers (≥20% disagreement). For instance, 78% of HCPs reported that the PPCT helps to better align the care to the child and family’s needs. However, this item did not become a facilitator as it was below the 80% cut off, but it could still influence the implementation process. This is the first study to evaluate the implementation of a PPCT by using a standardised survey, and we plan to use a qualitative approach in future studies to improve our understanding of PPCT implementation. Our studies can lead to the development of tailored implementations strategies for PPCTs to improve PPC both in the intramural and home settings.

## Conclusion

This is the first study on the implementation of a PPCT in the Netherlands. The study contributes to the current literature on implementation of PPCTs by using the innovative evaluation tool MIDI. To overcome the identified barriers, organisations need to establish working arrangements to efficiently collaborate with the PPCT. This study also found that implementing a PPCT can be impeded because of other changes occurring within the HCP’s own organisation. New PPCTs need protection and resources in their initial year to become experienced and qualified members of the PPCT and to allow other HCPs to experience the value of PPCTs in providing PPC. Positive experiences should be shared through presentations at grand rounds, conferences and paediatric palliative care network meetings. Also, involved HCPs should be given concrete details about support and responsibilities of the PPCT to ensure its successful implementation.

## Additional file

Additional file 1: **Table S1.** Determinants for implementing a paediatric palliative care team (PPCT) as measured by the Measurement Instrument for Determinants of Innovations (*n* = 71). Overview of the determinants for implementing a paediatric palliative care team as measured by the Measurement Instrument for Determinants of Innovations. (DOCX 24 kb)

## Abbreviations

AAP: American Academy of Pediatrics
HCP: Healthcare professional
IMPaCCT: International Meeting for Palliative Care in Children, Trento
IoM: Institute of Medicine
MIDI: Measurement Instrument for Determinants of Innovations
PPC: Paediatric palliative care
PPCT: Paediatric palliative care team
