# Supplementary material for: Barriers and facilitators to the implementation of a paediatric palliative care team
Source: BMC Palliat Care. 2018 Feb 12;17:23. doi: 10.1186/s12904-018-0274-8 (PMC5810030; doi:10.1186/s12904-018-0274-8)
Supplement: Supplementary file 1 — Table S1. Determinants for implementing a paediatric palliative care team (PPCT) as measured by the Measurement Instrument for Determinants of Innovations (n = 71). Overview of the determinants for implementing a paediatric palliative care team as measured by the Measurement Instrument for Determinants of Innovations. (DOCX 24 kb) [file 12904_2018_274_MOESM1_ESM.docx]

| **Additional file 1: Table S1.** Determinants for implementing a paediatric palliative care team (PPCT) as measured by the Measurement Instrument for Determinants of Innovations (*n*=71) | | | | | | | | | |
| --- | --- | --- | --- | --- | --- | --- | --- | --- | --- |
|  | | MIDI scale and items | **Mean** | **SD** | **Range** | **Disagree/totally disagree** | **Neutral** | **Agree/totally agree** | |
|  | |  |  |  |  | (%) | (%) | (%) | |
|  | | **Intervention (PPCT)** |  |  |  |  |  |  | |
| 1 | | Procedural clarity: the PPCT clearly indicates what I can expect from them | 3,82 | 0,93 | 1-5 | 12,7 | 11,3 | 76,1 | |
| 2 | | Correctness: the way PPCT operates is based on factually correct knowledge | 3,86 | 0,88 | 1-5 | 9,9 | 8,5 | **81,7** | |
| 3 | | Completeness: the information and materials provided by the PPCT is complete | 3,79 | 0,83 | 1-5 | 8,5 | 16,9 | 74,6 | |
| 4 | | Complexity: the PPCT is not too complex for me to use | 4,13 | 0,79 | 1-5 | 4,2 | 4,2 | **91,5** | |
| 5 | | Compatibility: the PPCT is compatible with values and working methods in place | 3,87 | 0,79 | 1-5 | 7,0 | 16,9 | 76,1 | |
| 6 | | Observability: the outcomes of using the PPCT are clearly observable | 3,99 | 0,82 | 1-5 | 7,0 | 12,7 | **80,3** | |
| 7 | | Relevance for patient: the PPCT is relevant for my patients | 4,20 | 0,80 | 1-5 | 5,6 | 7,0 | **87,3** | |
|  | | **User** |  |  |  |  |  |  | |
| 8a | | Personal benefits: the PPCT provides me time-savings | 3,56 | 1,05 | 1-5 | 16,9 | 19,7 | 63,4 | |
| 8b | | Personal benefits: the PPCT does not decrease my moments of contact with the family | 3,48 | 0,95 | 1-5 | 15,5 | 29,6 | 54,9 | |
| 8c | | Personal benefits: the PPCT helps me to become better aware of care needs of the family | 3,61 | 0,78 | 1-5 | 8,5 | 28,2 | 63,4 | |
| 8d | | Personal benefits: the PPCT helps me to feel more competent in providing PPC | 3,41 | 0,90 | 1-5 | 16,9 | 36,6 | 46,5 | |
| 8e | | Personal benefits: the PPCT does not perform tasks that I would rather do myself | 3,66 | 0,89 | 1-5 | 12,7 | 19,7 | 67,6 | |
| 8f | | Personal benefits: the PPCT does not perform tasks that I can better perform myself | 3,77 | 0,81 | 1-5 | 7,0 | 21,1 | 71,8 | |
| 8g | | Personal benefits: because of the PPCT it is clear to me who actively directs the care | 3,44 | 0,87 | 1-5 | 18,3 | 26,8 | 54,9 | |
| 8h | | Personal benefits: because of the PPCT my workload does not increase | 3,72 | 0,78 | 1-5 | 5,6 | 26,8 | 67,6 | |
| 8i | | Personal benefits: the PPCT helps me to attune my care for the family with other involved HCPs | 3,83 | 0,77 | 1-5 | 8,5 | 14,1 | 77,5 | |
| 8j | | Personal Benefits: the PPCT supports me in providing PPC | 3,99 | 0,75 | 1-5 | 5,6 | 11,3 | **83,1** | |
| 9a | | Outcome expectations: the PPCT helps to better attune the care to the needs of child and parents | 4,07 | 0,68 | 1-5 | 1,4 | 15,5 | **83,1** | |
| 9b | | Outcome expectations: the PPCT helps to improve the quality of care | 4,03 | 0,65 | 1-5 | 1,4 | 15,5 | **83,1** | |
| 9c | | Outcome expectations: the PPCT helps to improve the continuity of care | 3,99 | 0,71 | 1-5 | 2,8 | 16,9 | **80,3** | |
| 9d | | Outcome expectations: the PPCT helps to improve the coordination of care | 4,00 | 0,74 | 1-5 | 2,8 | 18,3 | 78,9 | |
| 9e | | Outcome expectations: the PPCT helps parents not to feel abandoned | 4,10 | 0,64 | 1-5 | 1,4 | 11,3 | **87,3** | |
| 9f | | Outcome expectations: the PPCT helps to decrease the number of hospitalisations of the child | 3,59 | 0,69 | 1-5 | 2,8 | 43,7 | 53,5 | |
| 9g | | Outcome expectations: the PPCT helps to early plan the care | 3,65 | 0,63 | 1-5 | 2,8 | 35,2 | 62,0 | |
| 9h | | Outcome expectations: the PPCT helps parents to continue caregiving at home | 3,93 | 0,64 | 1-5 | 2,8 | 15,5 | **81,7** | |
| 10 | | Professional obligation: I feel it as my responsibility to collaborate with the PPCT | 4,31 | 0,73 | 1-5 | 2,8 | 7,0 | **90,1** | |
| 11 | | Patient satisfaction: patients/parents are satisfied when the PPCT is involved | 3,97 | 0,72 | 1-5 | 2,8 | 14,1 | **83,1** | |
| 12a | | Patient cooperation: patients/parents generally cooperate when the PPCT is involved | 3,92 | 0,67 | 1-5 | 2,8 | 18,3 | 78,9 | |
| 12b | | Professional cooperation: HCPs generally cooperate when I collaborate with the PPCT | 3,89 | 0,60 | 1-5 | 1,4 | 19,7 | 78,9 | |
| 14 | | Descriptive norm: proportion of colleagues in own work field that will work together with the PPCT | 6,06 | 1,01 | 1-7* | 1,4 | 2,8 | **95,8** | |
| 15a | | Normative beliefs: parents expect me to collaborate with the PPCT | 3,70 | 0,68 | 1-5 | 0,0 | 42,3 | 57,7 | |
| 15b | | Motivation to comply: I find the PPCT opinion of parents important | 4,06 | 0,65 | 1-5 | 2,8 | 9,9 | **87,3** | |
| 16 | | Self-efficacy: I am able to call in the PPCT in time | 3,68 | 0,69 | 1-5 | 4,2 | 32,4 | 63,4 | |
| 17a | | Knowledge: the PPCT can directly be involved at time of the diagnosis of a LLD/LTD | 1,72 | 0,94 | 1-3 | 4,2 | 33,8 | 62,0 | |
| 17b | | Knowledge: I have enough information to call in the PPCT as intended | 3,61 | 0,93 | 1-5 | 14,1 | 23,9 | 62,0 | |
| 18 | | Awareness of content: I am informed on the content of the PPCT | 3,37 | 0,88 | 1-5 | 15,5 | 26,8 | 57,7 | |
|  | | **Organisation** |  |  |  |  |  |  | |
| 19 | | Formal ratification by management: there are formal arrangements relating the use of the PPCT | 1,66 | 0,79 | 1-3** | **53,5** | 19,7 | 26,8 | |
| 20 | | Replacement when I leave: colleagues are prepared to take over my collaboration with the PPCT | 3,51 | 0,91 | 1-5 | 14,1 | 22,5 | 63,4 | |
| 23 | | Time available: there is enough time available to collaborate with the PPCT in my daily work | 3,62 | 0,82 | 1-5 | 9,9 | 21,1 | 69,0 | |
| 26 | | Unsettled organisation: other changes going on that influence implementation of the PPCT | 1,72 | 0,45 | 1-2*** | **28,2** |  | 71,8 | |
| 27a | | Information accessible: easy to receive information about patients/parents from the PPCT | 3,86 | 0,70 | 1-5 | 4,2 | 15,5 | **80,3** | |
| 27b | | Information accessible: easy to find the information provided by the PPCT | 3,45 | 0,84 | 1-5 | 15,5 | 26,8 | 57,7 | |
| 27c | | Information accessible: easy to contact the case manager of the PPCT | 3,91 | 0,67 | 1-5 | 2,8 | 18,3 | 78,9 | |
| 28 | | Feedback to user about collaboration with the PPCT: regular feedback is provided | 3,44 | 0,89 | 1-5 | 14,1 | 32,4 | 53,5 | |
|  | | **Socio-political context** |  |  |  |  |  |  | |
| 29 | | Regulations: the PPCT fits well in existing guidelines | 3,76 | 0,73 | 1-5 | 4,2 | 28,2 | 67,6 | |
| Numbers in **bold** represent a HCP-reported barrier (≥ 20% disagrees/totally disagree) or facilitator (≥ 80% agrees/totally agrees). | | | | | | | |  |  |
| *: Answer categories were divided in 1 'not a single colleague, almost no colleague, a minority', 2 'half of colleagues', 3 'a majority, almost all colleagues, all colleagues'.  **: Answer categories were divided in 1 ’no’, 2 ‘I don’t know’, 3 ‘yes’.  ***: Answer categories were divided in 1 ‘yes’ and 2 ‘no’.  HCP: health care professional; LLD: life-limiting disease; LTD: life-threatening disease; PPCT: paediatric palliative care team. | | | | | | | |  |  |
